# Moderating Effects of Parental Feeding Practices and Emotional Eating on Dietary Intake among Overweight African American Adolescents

**DOI:** 10.3390/nu13061920

**Published:** 2021-06-03

**Authors:** Mary Quattlebaum, Dawn K. Wilson, Allison M. Sweeney, Nicole Zarrett

**Affiliations:** 1 Department of Psychology, University of South Carolina, Columbia, SC 29208, USA; mjq@email.sc.edu (M.Q.); zarrettn@mailbox.sc.edu (N.Z.); 2 College of Nursing, University of South Carolina, Columbia, SC 29208, USA; sweeneam@mailbox.sc.edu

**Keywords:** parental feeding practices, emotional eating, dietary intake, adolescent, African Americans

## Abstract

This study examined the effects of parental feeding practices and adolescent emotional eating (EE) on dietary outcomes among overweight African American adolescents. Based on Family Systems Theory, it was hypothesized that parental feeding practices, such as parental monitoring and responsibility, would buffer the effects of EE on poor dietary quality, whereas practices such as concern about a child’s weight, restriction, and pressure-to-eat would exacerbate this relationship. Adolescents (N = 127; *M*age = 12.83 ± 1.74; *M*BMI% = 96.61 ± 4.14) provided baseline data from the Families Improving Together (FIT) for Weight Loss trial and an ancillary study. Dietary outcomes (fruit and vegetables (F&Vs), energy intake, sweetened beverage, total fat, and saturated fat) were assessed using random 24-h dietary recalls. Validated surveys were used to assess adolescent-reported EE and parental feeding practices. Results demonstrated a significant interaction between EE and parental monitoring (adjusted analyses; *B* = 0.524, *SE* = 0.176, *p* = 0.004), restriction (*B* = −0.331, *SE* = 0.162, *p* = 0.043), and concern (*B* = −0.602, *SE* = 0.171, *p* = 0.001) on F&V intake; under high monitoring, low restriction, and low concern, EE was positively associated with F&V intake. There were no significant effects for the other dietary outcomes. These findings indicate that parental feeding practices and EE may be important factors to consider for dietary interventions, specifically for F&V intake, among overweight African American adolescents.

## 1. Introduction

The prevalence rate of adolescent obesity in the US is 20.6% [1], with higher rates of overweight or obesity shown among African American adolescents (40%) compared to their White peers (31%) [2]. Adolescent obesity has largely been attributed to physical inactivity, sedentary behaviors, and increased intake of energy-dense foods [3]. Problematic eating behaviors, such as emotional eating—the tendency to overeat in response to negative emotions—may also contribute to adolescent obesity [4]. Specifically, emotional eating has been linked to unhealthy dietary intake, including increased intake of energy-dense foods and sweetened beverages and reduced fruit and vegetable (F&V) intake, among diverse adolescent samples (e.g., African American and Latino) [5,6,7,8]. Adolescent African Americans have shown higher rates of emotional eating compared to their White peers, and thus may be at greater risk for potential weight gain [9]. Emotional eating has also been associated with poor psychosocial outcomes (e.g., lower quality of life, mental health concerns, and body dissatisfaction) [10,11], and growing evidence demonstrates an association between adolescent emotional eating and dietary intake and parental feeding practices (parental behaviors to influence their child’s food intake or eating behaviors) [12,13]. Thus, further examination of parental feeding practices may elucidate the relationship between emotional eating and poor dietary quality, which may be particularly important to African American adolescents.

African American adolescents and their families are disproportionately exposed to various social–environmental conditions (e.g., poverty, neighborhood disorder, and lack of access to healthy foods) that may contribute to a greater risk of emotional eating and related health consequences [14,15,16,17]. African Americans may cope with these social environmental factors and chronic stress by engaging in emotional eating, which may be due to a lack of resources [18,19]. For example, one study showed that having limited access to high-dietary-quality foods in homes and neighborhoods was associated with greater consumption of high-fat, high-sugar foods within an adolescent sample (90.7% African American) [14]. Thus, African American adolescents who experience emotional eating may be at risk of consuming more poor-dietary-quality foods readily available in their home and neighborhood environment. In addition, parental modeling may impact adolescents’ eating patterns. In the context of the family system, parents may model poor eating habits, such as emotional eating in response to stress [20]. Thus, understanding parenting moderators of emotional eating on dietary outcomes may be particularly important among high-risk overweight African American adolescents.

Family Systems Theory (FST) proposes that supportive, nurturing family interactions and positive parenting behaviors (warmth and communication) are important for promoting healthy development in adolescence, such as nutritious eating habits [21,22]. Parenting styles, including authoritative (high responsiveness and high demandingness) and authoritarian (low responsiveness and high demandingness), have shown important associations with adolescents’ eating behaviors and dietary intake [23]. Specifically, authoritative practices have been linked to higher F&V intake in children and adolescents [24], whereas authoritarian practices have been related to lower F&V intake [25]. In line with FST, parental feeding practices, such as monitoring a child’s eating (tracking a child’s eating) and responsibility (perception of parental responsibility for child’s eating), have been associated with reduced sweetened beverage intake and less emotional eating in children and adolescents [26,27]. In contrast, more restrictive parental feeding practices, including restricting a child’s access to foods, concern about a child’s weight, and pressure-to-eat have been linked to high-fat, high-sugar intake and high rates of emotional eating [28,29]. Based on FST, parental feeding practices, such as monitoring and responsibility, may help facilitate a supportive home climate, which may buffer the negative effects of adolescent emotional eating on dietary outcomes [30].

Few previous studies have examined parenting factors (parenting styles and feeding practices) in relation to adolescent dietary intake among solely African American families, and of the studies to date, there have been inconsistent results. Specifically, some research has shown that restrictive parental feeding practices have been related to greater F&V intake among low-income, predominantly African American children (43% African American) [31]. Furthermore, prior studies have shown that more demanding parenting practices (e.g., restriction and pressure-to-eat) were associated with increased self-regulation among African American adolescents, particularly among low-income families [32,33]. Conversely, other studies have shown that authoritative parental feeding practices are related to higher dietary quality among low-income minority children and adolescents (38% African American sample) [34]. More recent investigations by our group that included a sample of African American adolescents demonstrated that parental responsiveness was associated with high dietary self-efficacy for eating healthy [35]. Furthermore, another analysis showed that for African American adolescents who perceived higher parental pressure-to-eat, parental stress was associated with higher adolescent body mass index (BMI) [36]. The current study expands on past research by examining the moderation effects of parental feeding practices (both responsiveness and restrictive parenting practices) and adolescents’ emotional eating on dietary outcomes among high-risk, overweight African American adolescents.

Few studies have examined the moderating effects of parental feeding practices and emotional eating on dietary outcomes among African American adolescents; however, studies on adolescents in general show the relevance of this research. A recent study examined the moderating role of parental feeding practices and adolescent reward sensitivity on dietary intake (sugar-sweetened beverages and snack foods (categorized as healthy or unhealthy)) among Flemish adolescents [37]. Reward sensitivity is recognized as an individual’s responsiveness to reward cues [38] and has been associated with greater emotional eating, poor dietary habits, and risk for overweight [39,40,41]. Van Lippevelde et al. [37] found that with greater restriction and pressure-to-eat, for adolescents with high (vs. low) reward sensitivity there was a positive association with high-fat, high-sugar snack intake (e.g., cookies, pastries, fries, etc.). Thus, parental feeding practices, such as restriction and pressure-to-eat, exacerbated consumption of sugar and fat intake among adolescents with high reward sensitivity. This prior study did not include an ethnically diverse adolescent sample, and thus the present study examined the moderating effects of parental restriction and pressure-to-eat on emotional eating in predicting African American adolescents’ dietary intake.

Several other studies on inhibition and loss of control (LOC) eating also provide relevant information on moderated effects similar to those proposed in our study. For example, one study examined the interactive effects of parental feeding practices and preadolescent impulsivity (i.e., deficits in inhibition or control) on emotional eating among predominantly White preadolescents [42]. Farrow [42] found that under low or average, but not high, levels of parental monitoring of a child’s eating, preadolescent impulsivity was positively associated with emotional eating. Additional studies have examined the role of parental feeding practices in relation to child and adolescent LOC eating, which is recognized as a feeling of loss of control while eating, regardless of the amount of food consumed [43]. LOC eating is often paired with negative emotions and increased intake of energy-dense foods and has been shown to coincide with emotional eating behaviors, particularly among overweight youth [44,45,46,47]. Given the parallels between emotional eating and LOC eating, including negative affect, risk for poor-dietary-quality intake, and weight gain, this construct may be particularly relevant to the present study. A recent study assessed the interactive effects of parental feeding practices and adolescent weight status on LOC eating among adolescents [48]. The study findings indicated that for high maternal restriction of adolescent’s eating, weight status was positively associated with LOC eating. Thus, the impacts of restrictive parental feeding practices on LOC eating may be particularly exacerbated among adolescents with overweight or obesity. In sum, these studies highlight the important moderating role of parental feeding practices on problematic eating behaviors tied to negative emotions, particularly among high-risk adolescents. This research is limited, however, in that few past studies have examined these relationships among overweight African American adolescents.

The purpose of the current study was to examine the moderating effects of parental feeding practices on adolescent emotional eating in predicting dietary outcomes (F&V, energy intake, sweetened beverage, total fat, and saturated fat) among overweight African American adolescents in the Families Improving Together (FIT) for Weight Loss Trial [49,50]. Based on FST and previous research [37,42], it was hypothesized that parental feeding practices, including monitoring a child’s eating and perceived parental responsibility, would buffer the association between adolescent emotional eating and poor dietary intake, such that higher-dietary-quality outcomes were more likely (higher F&V intake, lower energy intake, sweetened beverage, total fat, and saturated fat). Conversely, it was hypothesized that parental feeding practices, such as restriction of a child’s eating, concern about a child’s weight, and parental pressure-to-eat, would exacerbate the association between adolescent emotional eating and dietary intake, such that lower-dietary-quality outcomes were more likely (lower F&V, higher energy intake, sweetened beverage, total fat, and saturated fat).

## 2. Materials and Methods

### 2.1. Participants

The current study included 127 African American parent-adolescent dyads from the FIT Weight Loss trial [49,50]. The participants also took part in an ancillary study, the Understanding Heredity and the Environment in African American Risk of Hypertension (HEART) study [51], which assessed stress and emotional eating in adolescents. Adolescents were eligible for participation if they (1) identified as African American, (2) were overweight or obese (BMI ≥ 85th percentile), (3) were between 11–16 years old, (4) had internet access, and (5) had a parent or guardian willing to participate in the study. Adolescents were excluded from the study if they had a medical or psychiatric condition that might interfere with physical activity or dietary habits, were taking any medications that could impact their weight or appetite, or if they were currently enrolled in another structured weight-loss program. Participants residing in Columbia, SC, were recruited through local clinics, schools, and community centers (i.e., churches and recreational centers) [52].

### 2.2. Procedures

The purpose of the FIT trial was to evaluate the efficacy of a motivational plus family-based weight-loss intervention versus a comprehensive health education program on reducing BMI in overweight and obese African American adolescents and their caregivers [49] (ClinicalTrials.gov ID#: NCT01796067). Baseline assessments were completed over a 2-week orientation period before starting the intervention. All FIT participants were also invited to complete the HEART study to further understand participants’ stress measures, including emotional eating. On average, participants completed FIT and HEART baseline visits within approximately two months of each other. The current study only evaluated the baseline data from both the FIT trial and HEART study. The FIT trial did not target emotional eating behaviors, and thus we do not expect there to be significant differences in emotional eating responses among the participants that completed their baseline visit at pre-intervention versus during the intervention. Moreover, prior studies utilizing data from the FIT trial and HEART study did not show significant effects when examining treatment effects in relation to time of measurement for a variable that was collected in the HEART study [36]. Adolescents and their parents provided written informed assent and consent, respectively, prior to participation in the study. Both studies were approved by the University of South Carolina Institutional Review Board. After completing study procedures, participants were compensated with $20 for their baseline assessment visit. Additional details regarding study design and procedures for the FIT trial are available [49].

### 2.3. Measures

#### 2.3.1. Demographic Information

Demographics were collected on adolescent age, adolescent sex, parent education, annual household income, parent marital status, and number of children under 18 years old living at home.

#### 2.3.2. Anthropometrics

Adolescent and parent height and weight were measured by a trained research assistant with a Shorr height board and SECA 880 digital scale, respectively. Height was measured in centimeters and weight was measured in kilograms. Two measurements of height and weight were collected, and a third measurement was taken if there was a difference greater than 1.0 cm or 0.5 kg between the first two measurements. BMI was calculated with an average of the height and weight measurements based on the Center for Disease Control (CDC) for adolescents and adults, respectively. Parent BMI values were used as a covariate in the current study.

#### 2.3.3. Emotional Eating

Emotional eating was assessed with the Three-Factor Eating Questionnaire—Revised 18-item (TFEQ-R18), which is a revised version of the 52-item Three-Factor Eating Questionnaire (TFEQ). The TFEQ-R18 includes eighteen total items that assess emotional eating, uncontrolled eating, and cognitive restraint. The current study assessed emotional eating specifically. Emotional eating items (3 items) included statements such as, “When I feel anxious, I find myself eating”. Responses are provided with a 4-point Likert scale from 1 (definitely true) to 4 (definitely false). Higher scores indicate greater emotional eating. This questionnaire has demonstrated good factor structure and construct validity among adolescents [53]. Prior studies suggest that this measure has internal consistency, with acceptable Cronbach’s alpha coefficients ranging from 0.77 to 0.84 [54]. The current study also demonstrated internal consistency, with a Cronbach’s alpha coefficient of 0.81.

#### 2.3.4. Dietary Outcomes

Adolescent dietary outcomes were collected using three random 24-h dietary recalls conducted with a registered dietician, which has been shown to be a valid measure [55]. It is the gold standard to conduct three 24-h dietary recalls to determine dietary intake in adolescents [56,57]. The telephone-administered recalls were completed on two weekdays and one weekend day. Adolescents were provided instructions at their FIT baseline visit on how to properly estimate portion sizes. During the recall, participants were asked to describe the type and amount of food they had eaten the day before. Daily F&V (with fried F&V items removed) and sweetened beverage intake (servings), energy intake (kilocalories), total fat and saturated fat intake (grams) were estimated, and each outcome was averaged from the completed recalls for the current study.

#### 2.3.5. Parental Feeding Practices

The Child Feeding Questionnaire (CFQ) was used to measure adolescent-reported parental feeding practices. The phrasing of the items was revised to reflect adolescent’s perspective of their parent’s feeding practices, as this measure is typically completed by the parent. Prior studies have shown that this is a valid approach to assess adolescents’ perception of parental feeding practices [42]. This questionnaire included 21 items that assessed five subscales: perceived parental responsibility, parental concern about a child’s weight, parental restriction, parental monitoring, and parental pressure-to-eat. Responses are captured with a 5-point Likert scale to determine the frequency of feeding practices (“never” to “always”) and the degree of agreement with a statement (“disagree” to “agree”). The current study included the following subscales: perceived parental responsibility (3 items, i.e., “How often is your parent responsible for deciding if you have eaten the right kind of foods?”), parental concern about a child’s weight (3 items, i.e., “How concerned is your parent about you dieting to maintain desirable weight?”), parental restriction (8 items, i.e., “Does your parent have to watch that you do not eat too much of your favorite foods?”), parental monitoring (3 items, i.e., “How often does your parent keep track of the sweets (candy, ice cream, cake, pies, and pastries) that you eat?”), and parental pressure-to-eat (4 items, i.e., “If you say, ‘I’m not hungry’, does your parent believe you should try to eat anyway?”). For each subscale, the scores were the sum of items included in the respective subscale. This measure has been shown to be a reliable scale across prior national studies [58]. The current study showed adequate reliability for this scale (monitoring, α = 0.91; responsibility, α = 0.66; restriction, α = 0.88; pressure-to-eat, α = 0.65; concern, α = 0.88).

### 2.4. Statistical Analysis

Analyses were conducted with IBM SPSS Statistics Version 26 and R-studio. The data were assessed for normality and outliers. Multicollinearity was examined to confirm that all VIF values were below 10 [59]. A hierarchical linear regression was utilized to examine the interaction between parental feeding practices (responsibility, monitoring, concern, restriction, and pressure-to-eat) and adolescent emotional eating predicting dietary outcomes (F&Vs, energy intake, sweetened beverages, total fat, and saturated fat). Regression analyses for each dietary outcome were run with and without covariates. Unadjusted regression analyses were conducted and did not change the overall results for each dietary outcome. Adjusted regression analyses were also run to test the interactions separately for each dietary outcome, which did not result in significant changes to the overall results. For unadjusted analyses, the first step of the model included the main effects of the predictors and the second step added the interaction terms; for adjusted analyses, a third step of the model added covariates. The third step of the model allowed us to examine whether adjusting for covariates impacted the model. Adjusted models included the following covariates: adolescent sex, adolescent age, parent education, parent BMI, and group treatment. These covariates have shown to be associated with adolescent dietary outcomes [60]. An omnibus F-test was utilized to evaluate effects with all interaction terms considered together in one model. This is a conservative statistical approach that has been utilized in prior studies assessing simultaneous interaction effects to decrease the likelihood of a type 1 error rate [61]. Separate models were constructed for each continuous dietary outcome. Adolescent sex (e.g., male vs. female), parent education (e.g., college vs. no college), and group treatment (e.g., intervention vs. control group) were dummy-coded for analyses.

Dietary data were cleaned prior to conducting analyses. Consistent with previous studies [62], to account for extreme scores in kcals, energy intake was corrected such that the minimum was set to 500 and the maximum was set to 5000, which resulted in the recoding of less than 2% of daily observations. Additionally, to account for extreme scores in total fat, saturated fat, and sweetened beverages, a Winsorizing approach was applied [63]. Specifically, extreme scores were recoded to three times the interquartile range, which allowed for the inclusion of all data, while also reducing the effect of potential outliers. This approach led to the recoding of less than 6% of observations across the dietary outcomes.

Scores for emotional eating and parental feeding practices were calculated by norming each item before summation. Thus, summed scale scores were transformed to z-scores to aid in analysis and interpretation of statistical models. F&V intake was indexed by summing the averages of daily F&V intake. Analyses were conducted on participants with at least one dietary recall, as not all participants were able to complete three recalls. Simple slope analyses plotted at 1 SD above and below the mean were conducted to decompose the significant interactive effects. The alpha level was set to *p* < 0.05. Overall, 23 participants were removed from analyses due to incomplete emotional eating and/or dietary recall data, resulting in a total of 127 participants with complete data that were included in analyses.

## 3. Results

### 3.1. Demographics and Anthropometrics

Table 1 provides the demographic information for the study sample. Of the 127 participants included in the current study, the sample of adolescents was predominantly female (65.4%), and the average age was 12.83 years old. The average BMI percentile for this sample of adolescents was 96.61, with similar rates of obesity among parents (BMI 37.46 ± 8.022). A total of 42.9% of parents had attended some college and the average household income ranged from $25k to $39k.

### 3.2. Correlational Analysis

Correlation analyses indicated that adolescent emotional eating was significantly correlated with F&V intake (*r* = 0.18). Furthermore, parent education was significantly associated with parent body mass index (*r* = −0.19). A number of the parental feeding practices were significantly correlated with each other in the expected direction; these are modest correlations ranging from *r* = 0.16 to 0.49 (Table 2).

### 3.3. Parental Feeding Practices and Emotional Eating on Fruit and Vegetable Intake

The moderating effect of parental feeding practices and adolescent emotional eating on F&V intake was assessed with unadjusted and adjusted hierarchical linear regression models (Table 3). Results of the adjusted model are presented. The first step of the regression model included only the main effects (parental feeding practices and emotional eating) and revealed an insignificant F change (*p* = 0.324). The second step of the model added all the interaction terms together in one model and yielded a significant F change (*p* = 0.001). The final step of the model added covariates and did not yield a significant F change from the second step of the model (*p* = 0.906), suggesting that the model did not change significantly with the addition of covariates. There was a significant main effect of emotional eating on F&V intake (*B* = 0.397, *SE* = 0.157, *p* = 0.013), such that greater emotional eating was associated with greater F&V intake. There was a significant interaction between parental feeding practices (monitoring of a child’s eating) and emotional eating on F&V intake (*B* = 0.524, *SE* = 0.176, *p* = 0.004). There was also a significant interaction between parental feeding practices (restriction, concern about a child’s weight, *B* = −0.331, *SE* = 0.162, *p* = 0.043; *B* = −0.602, *SE* = 0.171, *p* = 0.001, respectively) and emotional eating on F&V intake. Adjusted hierarchical linear regression analyses were also conducted with the inclusion of adolescent body mass index (zBMI) and removal of adolescent sex as covariates in the model. All original results for the final step of the adjusted model for F&V intake remained statistically significant (*p* < 0.05), except for a small change in one of the interaction effects (restriction, *p* = 0.055). Adolescent baseline zBMI was not a significant predictor in the adjusted model.

**Figure 1:**
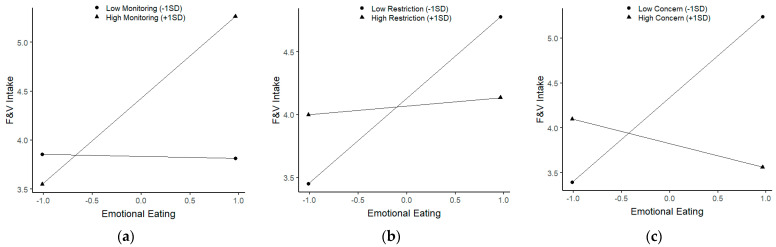
Significant interactions of parental feeding practices and emotional eating on fruit and vegetable intake. (**a**) The interaction between monitoring of a child’s eating and emotional eating on fruit and vegetable intake. (**b**) The interaction between restriction of a child’s eating and emotional eating on fruit and vegetable intake. (**c**) The interaction between concern about a child’s weight and emotional eating on fruit and vegetable intake.

Simple slopes analyses indicated that for parents with high monitoring of a child’s eating (*B* = 0.866, *SE* = 0.253, *p* = 0.001, Figure 1a), emotional eating was positively associated with F&V intake. Further, among parents with low restriction of a child’s eating (*B* = 0.672, *SE* = 0.212, *p* = 0.002, Figure 1b) or low concern about a child’s weight (*B* = 0.933, *SE* = 0.227, *p* = 0.000, Figure 1c), simple slopes analyses showed that emotional eating was positively associated with F&V intake. None of the other parenting factors interacted with emotional eating in predicting F&V intake.

### 3.4. Parental Feeding Practices and Adolescent Emotional Eating on Energy Intake (kcals)

The first and second step of the regression model for energy intake yielded an insignificant F change (*p* = 0.602, *R*^2^ = 0.037; *p* = 0.659, *R*^2^ = 0.064, respectively). The final step of the regression model for energy intake yielded a significant F-change (*p* = 0.024, *R*^2^ = 0.168); there was a main effect of parent education on adolescent energy intake (*B* = −297.480, *SE* = 99.501, *p* = 0.003), such that higher parent college education was associated with lower energy intake. There were no significant main effects or interactions with the parenting factors or emotional eating in the final step of the model.

### 3.5. Parental Feeding Practices and Adolescent Emotional Eating on Sweetened Beverage Intake

The final step of the regression model yielded insignificant F changes for sweetened beverage intake *(p* = 0.208).

### 3.6. Parental Feeding Practices and Adolescent Emotional Eating on Total Fat Intake

The final step of the regression model revealed insignificant F changes for total fat intake (*p* = 0.146).

### 3.7. Parental Feeding Practices and Adolescent Emotional Eating on Saturated Fat Intake

The final step of the regression model indicated insignificant F changes for saturated fat intake (*p* = 0.270).

### 3.8. Power Analyses

Power analyses were conducted using the R package “retrodesign” to determine the size of the interaction we had power to detect for the results presented in Table 3 [64]. For the five interactions, we had ≥80% power to find detectable differences between servings of F&V in the range of 0.62–1.94. Previous systematic reviews on changes in F&V suggest that an effect of 1 serving or greater is a large effect size [65,66]. Thus, in this study, we had power to detect a medium to large effect size for the interactions studied.

## 4. Discussion

The purpose of the current study was to evaluate the interaction between parental feeding practices and adolescent emotional eating on dietary outcomes among overweight African American adolescents. The results demonstrated that, with greater parental monitoring, emotional eating was positively associated with higher F&V intake. Further, for lower parental restriction and parental concern about a child’s weight, emotional eating was positively associated with F&V intake. No other findings were significant. Overall, these results demonstrated that higher levels of parental monitoring, as well as lower restriction and concern about a child’s weight, buffered the effect of emotional eating on poor-dietary-quality outcomes, as only F&V intake was associated with emotional eating. These results are consistent with our hypotheses that greater monitoring and less restrictive parental feeding practices may buffer the relationship between adolescent emotional eating and low F&V dietary intake in overweight African American adolescents.

In the current study, we found that, under high parental monitoring of the adolescent’s eating, adolescent emotional eating was positively associated with F&V intake. This finding of monitoring as a buffer of problematic adolescent eating habits aligns with prior research that shows that, under low (or average), but not high, levels of parental monitoring, a positive association between impulsivity and emotional eating has been demonstrated among predominantly White preadolescents [42]. Thus, reduced parental monitoring was related to greater emotional eating among youth who are at risk for impulsivity. Together, the current study and past studies [42] suggest that high parental monitoring may be a protective moderating factor for adolescent eating and diet-related outcomes (emotional eating and F&V intake). The current study, however, extends these findings to a high-risk group of overweight African American adolescents and examines the link between emotional eating and dietary quality (F&V intake). Considering that African American families face disproportionate social environmental chronic stressors that may place their families at greater risk for emotional eating compared to their White peers [15,16,17,18], these findings are particularly important and may inform future interventions.

The current study also found that, with lower levels of parental restriction of a child’s eating, adolescent emotional eating was positively associated with greater F&V intake. These findings suggest that reduced parental restriction may serve as a buffer of low F&V dietary intake in overweight African American adolescents. A previous study found that, under greater levels of parental restriction, reward sensitivity (i.e., response to reward cues) was positively associated with high-fat, high-sugar snack food intake (cookies, pastries, fries, etc.) among Flemish adolescents [37]. The current study is consistent with these past findings, as it indicates that less parental restriction may moderate adolescent emotional eating in improving dietary intake of F&Vs among overweight African American adolescents. While some prior research has shown that restrictive feeding practices are related to greater F&V intake within low-income samples of predominantly African American adolescents [31], the current findings and some prior studies with low-income minority children (38% African American) indicate that less restrictive feeding practices may be protective [34]. Notably, another study including a low-income sample of predominantly African American preadolescents (92% African American) found that parents with greater authoritarian and authoritative parenting predicted the highest adolescent dietary quality [67]. The authors proposed that parents may be utilizing helpful parenting practices from both approaches. It is plausible that some African American parents utilizing modest restrictive parental feeding practices are also demonstrating support and warmth through other parenting practices (authoritative parenting), resulting in better dietary quality among youth. This study and other investigators suggest that parenting practices among African American families may be better understood with the utilization of culturally appropriate measures and further examination of within-group differences [33]. Thus, additional research with African American adolescents is needed to identify the role of relevant contextual factors, individual differences, or other parent–adolescent dynamics on adolescent dietary quality.

Our results also indicated that, under low parental concern about a child’s weight, emotional eating was positively associated with F&V intake. Thus, decreased levels of parental concern may be protective of poor F&V dietary intake among African American adolescents. Few studies have examined parental concern about a child’s weight as a moderating factor of adolescent eating behaviors or diet; however, this parental feeding practice has been related to elevated adolescent stress-eating [68], weight status [69], and greater utilization of other restrictive parental feeding practices [70]. Further research is needed to elucidate the moderating effects of parental concern about a child’s weight on adolescent emotional eating and dietary intake and to examine the possible associations with weight stigma [71].

Of note, the results of this study showed a main effect of parent college education on adolescent energy intake, such that greater parent college education predicted healthier energy intake (reductions in kcals). This finding is important and aligns with prior research indicating that higher parental education levels are associated with lower intake of energy-dense foods among children and adolescents [72,73]. On average, the adolescents’ caregivers in our sample reported an educational status lower than a 4-year degree (58%), which may be important for understanding energy intake among our adolescent participants. Further studies should consider the relationship between parental education and parental feeding practices on adolescent dietary intake.

Our results indicated that the unadjusted models for energy intake, sweetened beverage intake, total fat intake, and saturated fat intake did not yield significant effects; however, the adjusted final model for energy intake did yield a significant main effect. Although sweetened beverage, energy, total fat, and saturated fat intake have been linked to parental feeding practices [74,75,76], limited studies have assessed emotional eating in relation to these dietary outcomes. In addition, there is growing research demonstrating that more than three dietary recalls are needed to show valid dietary assessments among adolescents [77]. However, emotional eating has been more closely linked to F&V intake, with some studies showing greater intake and others indicating lower intake of F&Vs [7,8]. More studies are needed to elucidate which dietary outcomes are related to adolescent emotional eating and further delineate how parenting factors may moderate emotional eating on understanding dietary outcomes. There may be individual differences in the type and amount of food that adolescents consume when eating in response to negative emotions. While some adolescents increase consumption of specific foods while engaging in emotional eating, others increase their overall intake of a variety of foods [7]. Thus, within our sample, the moderating effect of parental feeding practices may be more useful in examining emotional eating related to specific foods, such as F&V, rather than the overall amount or the nutritional makeup of food intake. It is also plausible that adolescents may be more willing to add more F&Vs to their diet than remove high-fat, high-sugar foods, but it will be important for longitudinal data to expand on the current research. Additional research on the family eating behaviors, such as family mealtime or parental emotional eating, may also inform the relationship between adolescent emotional eating and dietary intake.

This study has several limitations. First, it is a cross-sectional study, and causal inferences cannot be made, and we cannot confirm the direction of our effects. Longitudinal analyses are needed to better understand the influence of the home climate on adolescent emotional eating and dietary intake over time. Specifically, longitudinal data would provide insights into whether parental feeding practices elicit changes in emotional eating and dietary intake or if these problematic eating behaviors evoke certain parental feeding practices. Further, evaluating the development of emotional eating within the context of the home environment from childhood into adolescence will be informative, such as assessing how parents may model emotional eating. Moreover, understanding and intervening on emotional eating early in development through a family systems lens is critical to offset poor health trajectories into young adulthood. This study is also limited in that only three dietary recalls were administered which may result in unreliable dietary data [77] and thus may explain why only F&V intake was found to show significant interactions in the present study. Further, another limitation of this study may be that the current sample underreported their overall energy intake, given that our sample’s average energy intake is lower than typically reported in national studies with overweight adolescent samples [78]. Though it is the gold standard that diet-related studies collect three dietary recalls, research indicates that between 8 and 32 recalls are recommended for adequate reliability [77]. Further, our findings are limited given that the sample included overweight or obese African American adolescents from an urban area in the Southeast US, which may limit generalizability to normal-weight adolescents and non-minorities or families living outside the Southeast.

In summary, the findings in the current study suggest that, under high parental monitoring, as well as low restriction and concern about a child’s weight, emotional eating is related to greater F&V intake. Thus, parental feeding practices such as monitoring, as well as lower levels of restrictive practices, may be related to F&V dietary outcomes among African American adolescents. These findings are particularly noteworthy, considering the mixed literature regarding monitoring feeding practices versus restrictive feeding practices and adolescent eating behaviors within African American families [31,34]. Additional data are needed to further elucidate potential cultural differences in these parenting practices, including the influence of social environmental conditions and risk factors that are salient to African American families. Moreover, future studies are needed to better address how parenting factors or other relevant constructs may attenuate adolescent emotional eating and promote more adaptive coping strategies, such as eating higher-dietary-quality options (e.g., F&Vs). Given the notable influence of parental feeding practices on adolescent eating behaviors shown in our findings, it will also be important for future interventions to integrate a family systems approach.

## Figures and Tables

**Table 1:** Sample characteristics.

	Total (*N* = 127)
Age (years), M ± SD	12.83 ± 1.745
Race, %	
African American	100
Female, %	65.4
BMI Percentile (kg/m^2^ %), M ± SD	96.61 ± 4.142
Daily Energy Intake (kcals), M ± SD	1667.79 ± 510.652
Parent BMI (kg/m^2^), M ± SD	37.46 ± 8.022
Annual Household Income, %	
Less than 10k	11.9
10–24k	20.6
25–39k	27.0
40–54k	13.5
55–69k	8.7
70–84k	4.0
85k+	14.3
Parent Education, %	
9–11 years	3.2
12 years	12.7
Some college	42.9
4-year college	17.5
Professional	23.8
Marital Status, %	
Married	35.7
Separated	14.3
Divorced	20.6
Widowed	2.4
Never Married	19.8
In an unmarried couple	7.1
Number of Children at Home, %	
0	5.6
1–2	63.5
3–4	28.5
5–6	1.6
7	0.8

Note: M = mean; SD = standard deviation; BMI = body mass index, Avg. = average; k = thousand.

**Table 2:** Correlations among parental feeding practices, adolescent emotional eating, and adolescent dietary intake.

	1	2	3	4	5	6	7	8	9	10	11	12
1. Adolescent Age	-											
2. Adolescent Sex	0.05	-										
3. Treatment Group	−0.02	<0.01	-									
4. Parent BMI	−0.09	0.13	0.02	-								
5. Parent College	0.09	−0.16	−0.14	−0.19 *	-							
6. Emotional Eating	−0.08	−0.08	0.03	0.13	0.11	-						
7. Parental Responsibility	−0.29 **	0.05	−0.04	−0.002	0.01	0.13	-					
8. Parental Concern	−0.13	−0.13	−0.08	−0.09	0.16	0.14	0.40 **	-				
9. Parental Monitoring	−0.11	−0.06	−0.07	−0.11	0.15	−0.07	0.49 **	0.47 **	-			
10. Parental Restriction	−0.33 **	−0.09	−0.01	−0.02	0.01	0.18 *	0.41 **	0.34 **	0.46 **	-		
11. Parental Pressure-to-Eat	−0.17	−0.13	0.09	<0.01	−0.02	0.16	0.22 *	0.16	0.33 **	0.41 **	-	
12. Adolescent Fruit and Vegetable Intake	−0.06	−0.05	0.05	−0.01	0.07	0.18 *	0.02	−0.04	0.06	0.03	0.02	-

Note: * Indicates correlations significant with alpha criteria of *p* < 0.05; ** indicates correlations significant with alpha criteria of *p* < 0.01. Column headings correspond to row names.

**Table 3:** Hierarchical regression analyses assessing the interaction effects of parental feeding practices and adolescent emotional eating on adolescent fruit and vegetable intake.

Model		*B*	*SE*	*t*	*p*	*R* ^2^	Δ*R*^2^	ΔF Sig
1	Intercept	2.419	0.140	17.268	0.000 *	0.056	0.056	0.324
	Emotional Eating	0.361	0.152	2.378	0.019 *			
	Responsibility	−0.051	0.171	−0.298	0.766			
	Concern	−0.212	0.168	−1.262	0.209			
	Monitoring	0.280	0.187	1.497	0.137			
	Restriction	−0.027	0.167	−0.162	0.871			
	Pressure-to-Eat	−0.068	0.166	−0.411	0.682			
2	Intercept	2.589	0.139	18.679	0.000 *	0.206	0.150	0.001*
	Emotional Eating	0.395	0.151	2.613	0.010 *			
	Responsibility	−0.121	0.162	−0.746	0.457			
	Concern	−0.204	0.161	−1.261	0.210			
	Monitoring	0.236	0.178	1.328	0.187			
	Restriction	0.048	0.159	0.304	0.762			
	Pressure-to-Eat	−0.031	0.158	−0.195	0.846			
	EE*Responsibility	0.083	0.163	0.508	0.613			
	EE*Concern	−0.575	0.164	−3.507	0.001 *			
	EE*Monitoring	0.535	0.169	3.163	0.002 *			
	EE*Restriction	−0.343	0.154	−2.222	0.028 *			
	EE*Pressure-to-Eat	0.022	0.159	0.138	0.890			
3	Intercept	3.607	1.370	2.632	0.010 *	0.218	0.011	0.906
	Emotional Eating	0.397	0.157	2.525	0.013 *			
	Responsibility	−0.119	0.171	−0.695	0.489			
	Concern	−0.236	0.167	−1.411	0.161			
	Monitoring	0.256	0.186	1.376	0.172			
	Restriction	0.009	0.169	0.052	0.959			
	Pressure-to-Eat	−0.046	0.165	−0.278	0.781			
	EE*Responsibility	0.113	0.171	0.659	0.511			
	EE*Concern	−0.602	0.171	−3.528	0.001 *			
	EE*Monitoring	0.524	0.176	2.981	0.004 *			
	EE*Restriction	−0.331	0.162	−2.047	0.043 *			
	EE*Pressure-to-Eat	0.034	0.165	0.206	0.837			
	Adolescent Age	−0.051	0.087	−0.586	0.559			
	Adolescent Sex	−0.251	0.298	−0.841	0.402			
	Treatment Group	0.107	0.277	0.386	0.701			
	Parent BMI	−0.009	0.018	−0.501	0.617			
	Parent College	−0.021	0.296	−0.070	0.945			

Note: * Indicates a significant alpha criteria of *p* < 0.05. BMI = body mass index; EE = emotional eating. Models 1 and 2 include findings from the two steps of the unadjusted regression analyses; Model 3 includes the findings from the final step of the adjusted regression analyses.

## Data Availability

The data presented in this study are available upon request from the corresponding author. The data are not currently publicly available, as the outcome paper has not yet been published.
